# Affective evaluation of consciously perceived emotional faces reveals a “correct attribution effect”

**DOI:** 10.3389/fpsyg.2023.1146107

**Published:** 2023-05-25

**Authors:** Nicolas M. Brunet

**Affiliations:** ^1^Department of Psychology and Neuroscience, Millsaps College, Jackson, MS, United States; ^2^Department of Psychology, California State University, San Bernardino, San Bernardino, CA, United States

**Keywords:** affective priming effect, valence, misattribution effect, emotional faces, prime, target, prime duration, affect

## Abstract

The strength of the affective priming effect is influenced by various factors, including the duration of the prime. Surprisingly, short-duration primes that are around the threshold for conscious awareness typically result in stronger effects compared to long-duration primes. The misattribution effect theory suggest that subliminal primes do not provide sufficient cognitive processing time for the affective feeling to be attributed to the prime. Instead, the neutral target being evaluated is credited for the affective experience. In everyday social interactions, we shift our gaze from one face to another, typically contemplating each face for only a few seconds. It is reasonable to assume that no affective priming takes place during such interactions. To investigate whether this is indeed the case, participants were asked to rate the valence of faces displayed one by one. Each face image simultaneously served as both a target (primed by the previous trial) and a prime (for the next trial). Depending on the participant’s response time, images were typically displayed for about 1–2 s. As predicted by the misattribution effect theory, neutral targets were not affected by positive affective priming. However, non-neutral targets showed a robust priming effect, with emotional faces being perceived as even more negative or positive when the previously seen face was emotionally congruent. These results suggest that a “correct attribution effect” modulates how we perceive faces, continuously impacting our social interactions. Given the importance of faces in social communication, these findings have wide-ranging implications.

## Introduction

Semantic priming refers to the phenomenon where exposure to a stimulus, known as the prime, influences the response to a subsequent stimulus, the target. In typical experiments, participants classify objects into categories such as “living” or “inanimate.” The response time is faster when the target belongs to the same category as the prime, indicating that the prime influences perception. Fazio et al. (1986) found that the effect also applies to valence judgments. In other words, stimuli that follow a prime are evaluated faster when the valence is congruent, either positive or negative, compared with incongruent stimuli. Additionally, neutral stimuli can be shifted toward the valence of the prime. Interestingly, this effect is more robust when the prime is too short to be consciously perceived (Stapel and Koomen, 2005; Rieth and Huber, 2010; Barbot and Kouider, 2012).

One theory to explain this counterintuitive behavior is the misattribution theory, which suggests that longer primes allow for a more accurate link between the affective experience and its source, weakening the priming effect and lowering the likelihood of affect misattribution (Gawronski and Ye, 2014; Payne and Lundberg, 2014). However, it’s unclear if this also applies to face-only stimuli. Faces are a unique type of stimuli, and face recognition involves complex mechanisms across multiple brain regions, as illustrated by Zhen et al. (2013), who identified twenty-five regions that responded to face stimuli. Previous studies have found that affective priming biases perception when the preceding face is emotional and briefly displayed, whereas an adaptation bias modulates physical characteristics when displayed for a longer duration. It’s not clear if misattribution theory can explain affective priming effects observed in face-only stimuli.

Previous studies have found that subliminally priming a happy face can make surprised faces appear more positive compared with neutral or fearful faces, and that memory for surprised faces is facilitated up to 24 h after exposure to a 30-millisecond prime (Sweeny et al., 2009). However, few studies have investigated how long exposure to a consciously perceived face affects the perceived valence of the next face. A recent study (Zhang, 2020) found that participants who watched a short “funny” video clip evaluated faces as more positive compared with controls. Unfortunately, the study did not provide details about the video clip’s content.

The aim of this study is to investigate the impact of long-duration primes on affect perception using face-only stimuli. Rather than using the traditional approach of presenting stimuli as prime-target pairs, participants were asked to continuously rate faces displayed on a monitor one by one, drawn from three categories with neutral, negative, or positive valence, shown in a randomized order. To examine the effect of the preceding stimulus, the image viewed in trial n was considered the target, and the image viewed in trial n-1 was the prime. By alternating each image as both the target and prime, this approach offers opportunities to explore the effects of multiple prime-target combinations. This approach goes beyond the typical strategy of examining the impact of emotional primes on neutral targets to also examine how strongly emotional faces are influenced by primes.

To investigate whether different types of negative emotional faces have varying degrees of priming effects, the study employed blocks of trials consisting of “happy” and “neutral” faces, and either “fearful” or “angry” faces. Additionally, faces were presented either in half-profile view or frontal view to test whether potential priming effects would be weaker for the former due to the reduced perceived facial expression intensity reported in previous studies (Guo and Shaw, 2015; Sutherland et al., 2017; Surcinelli et al., 2022). The study also included a fifth block of stimuli where only the eyes and surrounding area of a face (either neutral, happy, or fearful) were visible, as eyes are an important source of social information (Marsh et al., 2005) and have been linked to activation in the amygdala in response to fearful expressions (Whalen et al., 2004). Moreover, stimuli showing widened eyes are easily linked to the emotion of fear (Smith et al., 2005), while crinkling around the eyes is associated with enjoyment smiles. The ability to recognize emotions from the eyes is thus high; this is especially the case in adults (Guarnera et al., 2017). The study aimed to test the following predictions: (a) a form of affective primacy effect will be observed despite long exposure to consciously perceived primes, (b) this effect, if observed, will be attenuated for faces seen in half-profile compared to those seen in frontal view, and (c) the effect will also be observed for stimuli where only the eyes and surrounding area are displayed.

## Materials and methods

### Subjects

In this study, a total of 28 undergraduate students with a mean age of 20.4 years (SD = 1.4) participated. Among them, 7 identified as male and 21 as female. Regarding ethnicity, 16 identified as white Hispanic or Caucasian, 11 as black or African American, and 1 as Asian. All participants were recruited from an introductory psychology course and received class credit in exchange for their participation. Every student provided informed consent, and none of them participated more than once. The sessions lasted approximately 30 min.

### Stimuli and experimental design

During the study, the participants were seated in front of a 19-inch Dell monitor, positioned 50 cm from their head, while 450 images were presented sequentially at the center of the screen (17^o^ × 23^o^ of visual angle). Their task was to evaluate the valence of each image on a scale of 1 (very positive) to 9 (very negative) using the keyboard. Once the participant evaluated the image, it disappeared from the screen, and a 1-s inter-trial interval, during which a cross was displayed at the center of the screen, followed before the next image appeared. The participants were instructed to respond as quickly and accurately as possible, and images not evaluated within 4 s disappeared from the screen. The experimental paradigm was created using Experiment Builder (SR Research).

**Figure 1:**
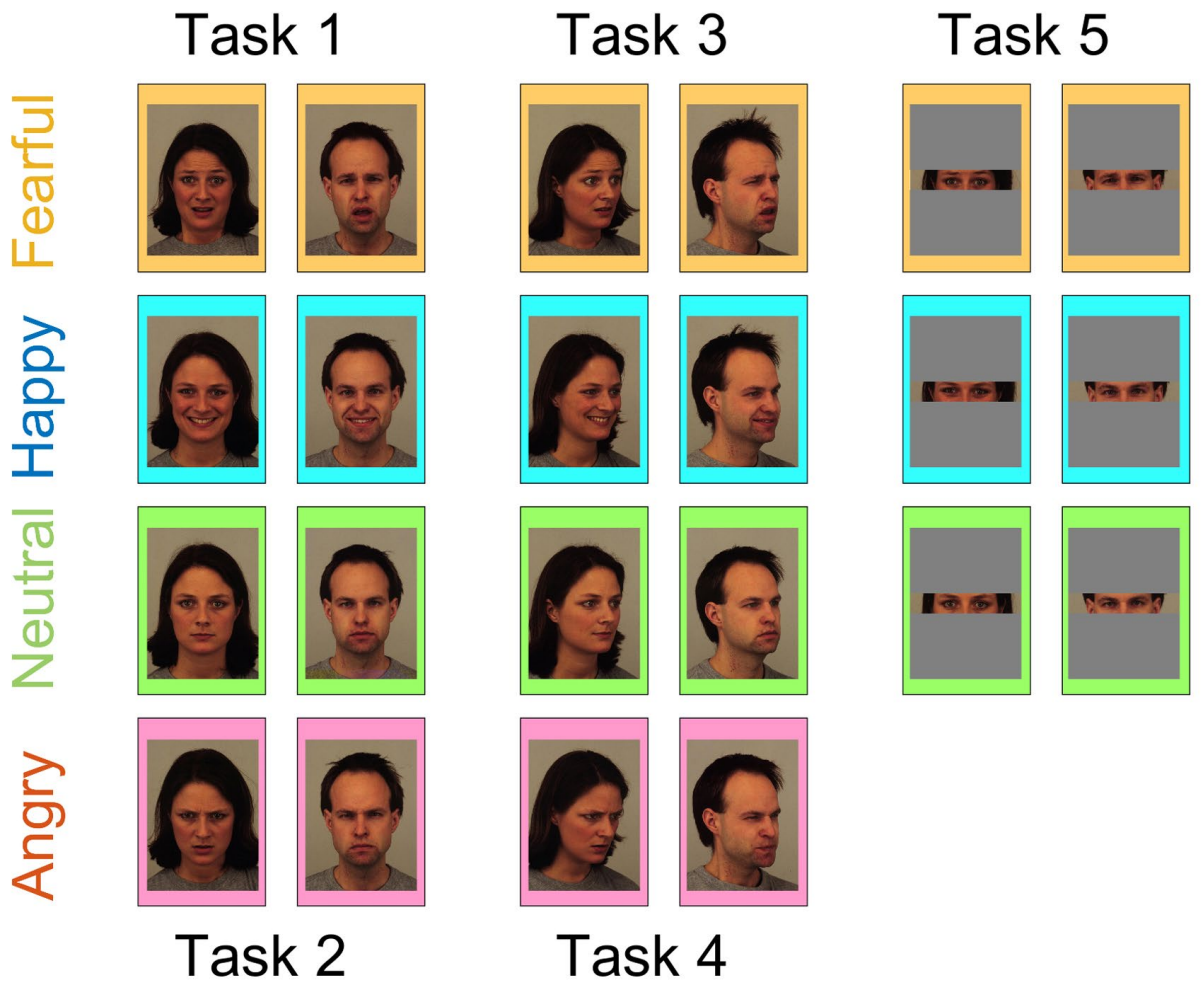
Example stimuli. Examples of the stimuli used in each of the five blocks. The faces were either presented in their entirety (Blocks 1–4) or cropped to show only the eyes and surrounding areas (Block 5). Fearful, happy, neutral, and angry faces are superimposed on orange, light blue, light green, and pink-filled rectangles, respectively, for clarity. As negative emotion condition, either “Fearful” (Blocks 1, 3, and 5; presented in solid rectangles) or “Angry” (Blocks 2 and 4; presented in dashed rectangles) was used. For each condition, we present an example of a male and a female face corresponding to models AM02 and AF01 from the KDEF database (Lundqvist et al., 1998). Reproduced with permission.

All of the stimuli, which included original (blocks 1 to 4) and manipulated (block 5) images, were selected from the Karolinska Directed Emotional Faces Dataset (Lundqvist et al., 1998), one of the most widely used databases for facial expressions. Block 1 included 90 images consisting of three different facial emotional expressions (“Happy,” “Neutral,” and “Fearful”), displayed by 15 males (models 1, 2, 4, 5, 6, 7, 8, 9, 10, 14, 17, 21, 22, and 27) and 15 females (models 1, 4, 5, 6, 9, 11, 12, 14, 15, 20, 21, 22, 24 and 29). Validation studies of the individual images, rated on an intensity and arousal scale, can be easily found (Goeleven et al., 2008). Block 2 was identical to block 1, except that the “Fearful” condition was replaced with an “Angry” condition. Blocks 3 and 4 were identical to blocks 1 and 2, respectively, except that they used left half-profile view faces instead of frontal view faces. Finally, block 5 was identical to block 1, except that each face was cropped to show only the eyes and surrounding area (see Figure 1 for examples of a set of male and female faces used for each block). Both blocks and the trials within each block were randomized for each participant.

### Analysis

Statistical analyses, including the computation of paired t-tests and repeated measures ANOVA, were conducted using Matlab/Simulink. The tests used and the determination of statistical significance are indicated in the text, figures, or figure captions as appropriate.

## Results

### Overall strategy

Participants rated the valence of each image on a scale ranging from 1 (very positive) to 9 (very negative). To make the display of results more intuitive, the scale was inverted, simply by subtracting the original value from 10. Each block consisted of 90 trials, with each trial’s target serving as the prime for the next trial, resulting in 89 primer-target pairs per block. Trials without a response and the subsequent trial were excluded from analysis. Since the stimuli consisted of three valence categories (positive, neutral, and negative), each numeric response could be categorized into one of nine primer-target conditions (e.g., positive–positive, positive-neutral). Averaging across conditions yielded nine values for each participant and each block; data used for the results presented in Figure 3 and Table 1.

**Figure 2:**
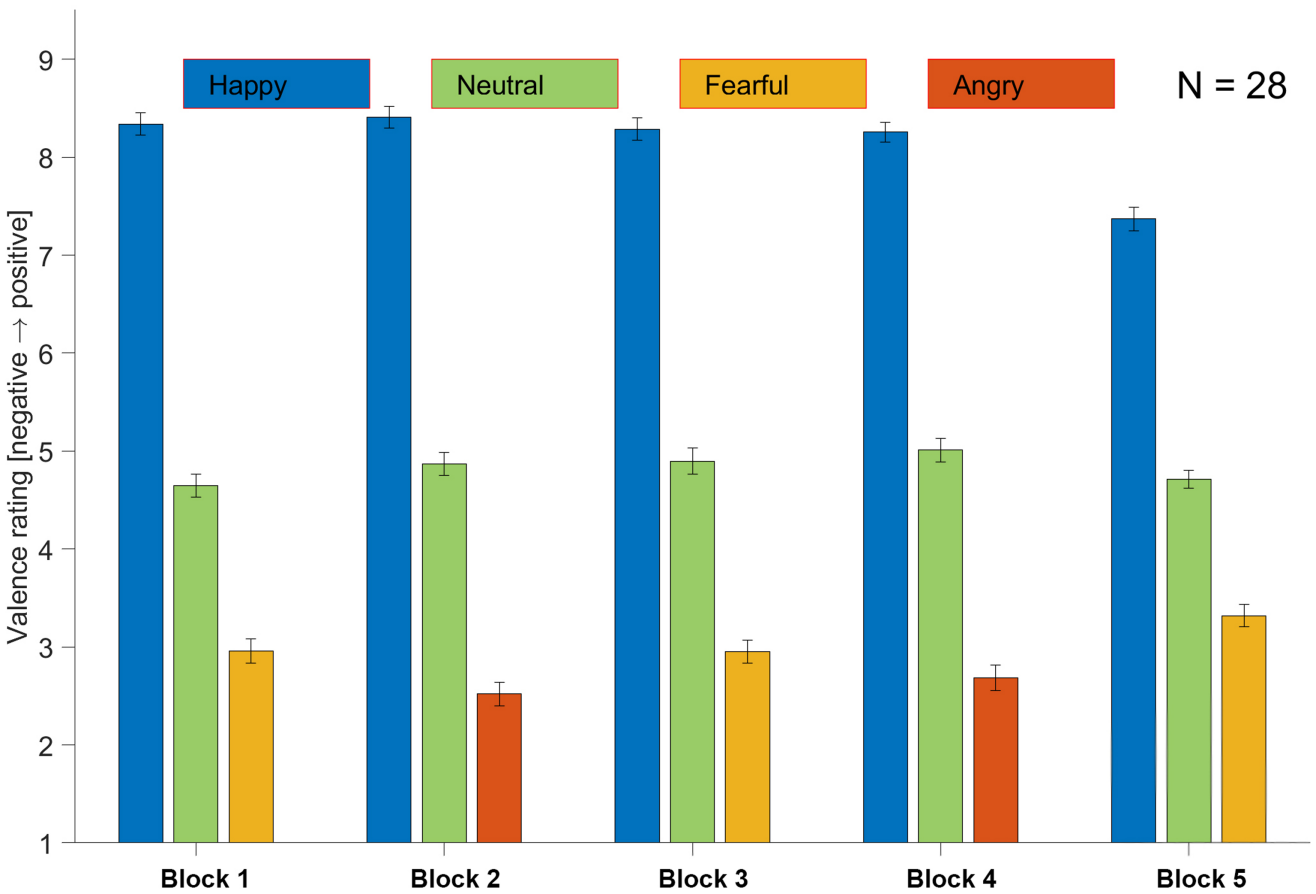
Task performance. The bar graph presents the task performance averaged across all 28 participants for each condition in each block. The stimuli used in Block 1 comprised “Happy,” “Neutral,” and “Fearful” faces displayed in the frontal view. The stimuli used in Block 2 were the same as those in Block 1, but with “Angry” faces replacing the “Fearful” faces. The stimuli in Blocks 3 and 4 were the same as those in Blocks 1 and 2, respectively, except that the faces were displayed in half-profile view. In Block 5, the same stimuli as in Block 1 were used, except each image was cropped to show only the eyes and surrounding area (see Figure 1 for examples of stimuli used in each block). Error bars indicate standard error.

**Figure 3:**
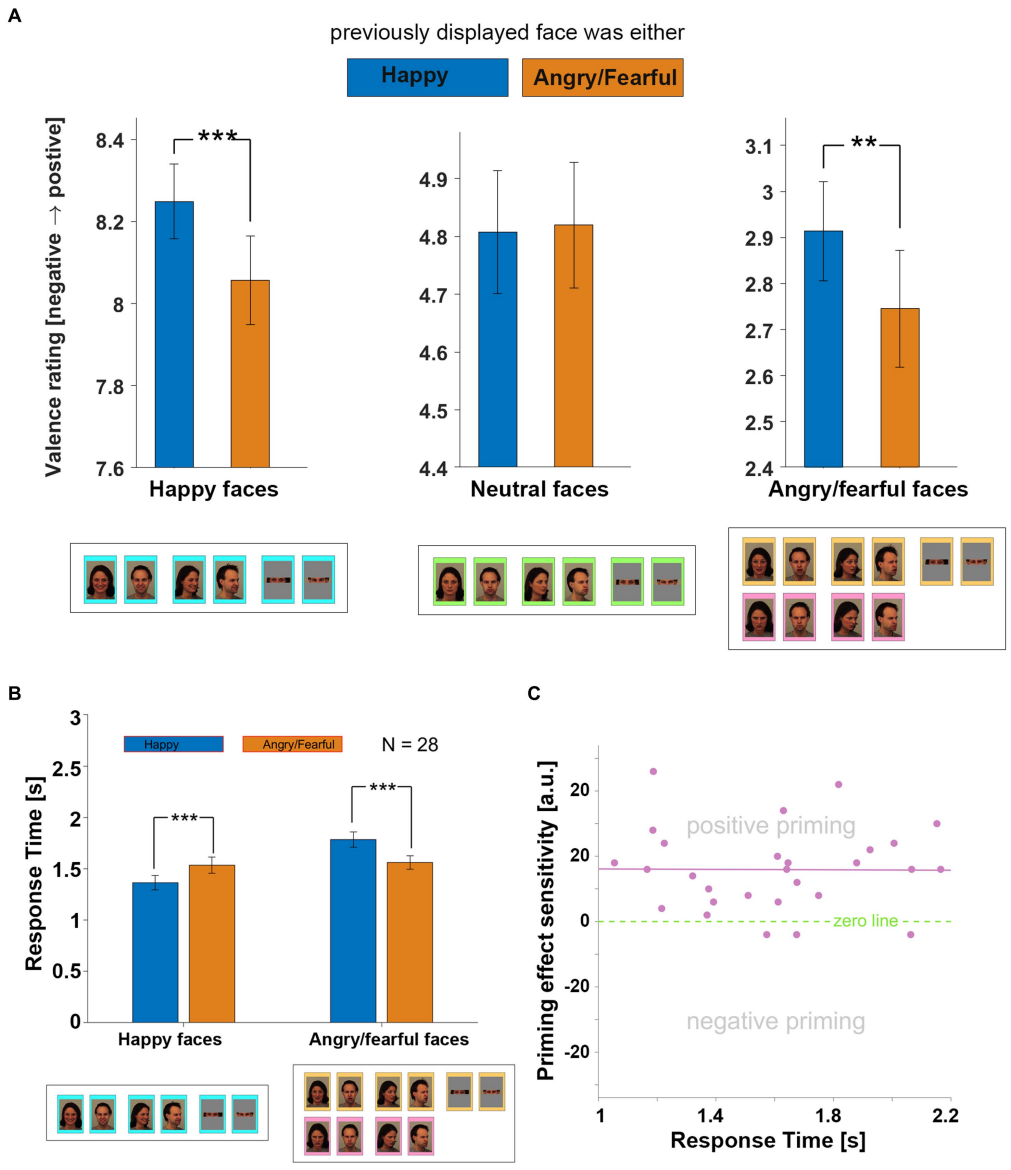
Affective priming effects. **(A)** This bar graph displays the average ratings given by all 28 participants in response to “Happy,” “Neutral,” and “Angry” or “Fearful” faces, with the color of the bars indicating the condition of the previously displayed image, either “Happy” (blue) or “Angry/Fearful” (orange). Results were obtained by averaging individual ratings across trials, all blocks, and across both negative-valence conditions (“Angry” and “Fearful”). The error bars represent standard error. Statistical significance was determined using a paired-sample *t*-test, and significance levels are indicated by one, two, or three asterisks, respectively representing *p*-values of equal to or less than 0.05, 0.01, or 0.001. For pairs of bars lacking stars, no significance was observed. **(B)** This bar graph displays the average reaction times of all 28 participants in response to “Happy” (left group) and “Angry” or “Fearful” faces (right group). The color of the bars indicates the condition of the previously displayed image, either “Happy” (blue) or “Angry/Fearful” (orange). **(C)** This scatterplot displays the sensitivity of the observed priming effect for each of the 28 participants, with each dot representing the data for one participant. The x-coordinate corresponds to the average response time across all trials, while the y-coordinate corresponds to the sensitivity of the observed priming effect for that individual. The sensitivity value was obtained through a specific method, which is explained in the text. Dots above and below the green zero-bar represent individuals exhibiting positive and negative priming effects, respectively. A least-squares line is shown in purple. The illustrated male and female faces, below panels (A,B), correspond to models AM02 and AF01 from the KDEF database (Lundqvist et al., 1998). Reproduced with permission.

**Table 1:** Affective ratings of emotional faces per block and averaged across all blocks.

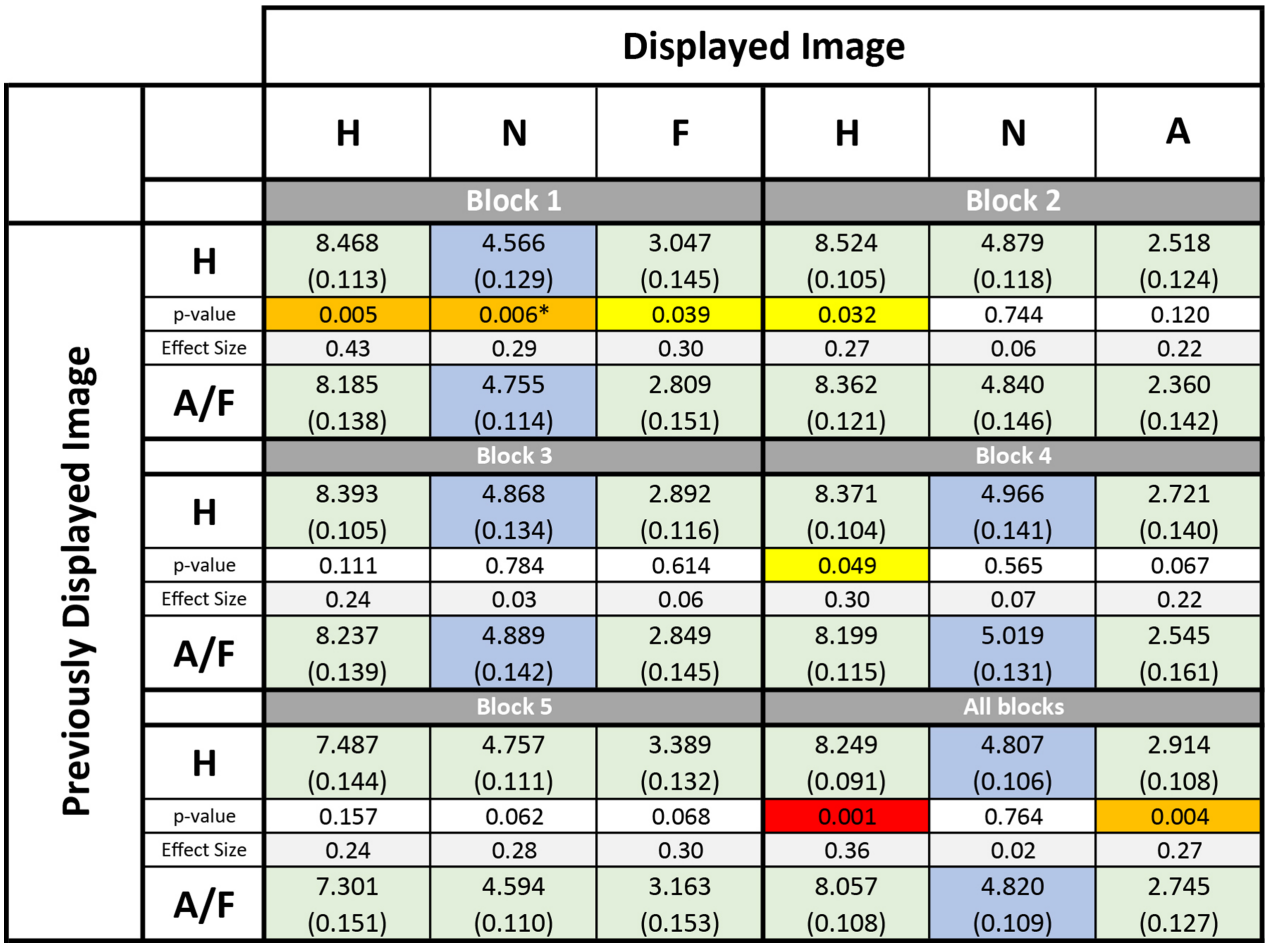

The results are presented for each experimental block (blocks 1 to 5) separately, and averaged across all blocks. Negative affect conditions, namely “Fearful” (used for blocks 1, 3 and 5) and “Angry” (used for blocks 2 and 4), were considered as one condition. For each block, there are three columns, reporting the affective ratings for each of the three different categories of targets: “Happy” (indicated with “H”), “Neutral” (indicated with “N”), and “Angry” or “Fearful” faces (indicated with “A/F”). The results were obtained by averaging across all trials of a given condition and then across all 28 participants. Standard errors are reported between brackets.

The rows labeled “H” and “A/F” correspond to the prime, the previously displayed image, which was either a “Happy” face or an “Angry” or “Fearful” face. Trials where the prime was a “Neutral” face were not considered for this analysis. *p*-values, obtained using a paired-sample *t*-test, and effect size values (Cohen’s *d*) are squeezed between each pair of measurements.

Pairs of values indicating a positive valence priming effect (as expected and hypothesized) are shaded in green, while pairs of values suggesting a negative priming effect are shaded in blue. For clarity, we used the following color code: *p*-values (1) above 0.05 are not shaded; (2) equal to or lower than 0.05 but higher than 0.01 are shaded in yellow; (3) equal to or lower than 0.01 but higher than 0.001 are shaded in orange; and (4) equal to or lower than 0.001 are shaded in red. Note that one of the reported *p*-values (marked with an asterisk) suggests significance for a negative, rather than a positive affective priming effect.

### Task performance

By averaging the behavioral results per condition (Figure 2), it is evident that participants accurately differentiated between the different conditions (“Fearful” or “Angry,” “Happy,” and “Neutral”) across all blocks, regardless of whether the faces were displayed in frontal (blocks 1 and 2) or half-profile (blocks 3 and 4) view. Despite the increased difficulty, this trend persisted in block 5, where only a partial view of the face (eyes and surrounding area) was presented.

### Affective priming effect

When averaging across all 5 blocks and all participants, it was found that valency ratings of both “Happy” faces and “Angry/Fearful” faces (Figure 3A) were affected by the previously displayed image. Specifically, “Happy” faces were perceived as more positive when the previous face was also “Happy”; conversely, “Angry” or “Fearful” faces were rated as more negative when the previous face was also “Angry” or “Fearful,” respectively. No such effect was observed for “Neutral” faces (Figure 3A). The response time, averaged across all participants, was also significantly faster when the valence of the face to be evaluated matched that of the previously displayed face (Figure 3B), consistent with the valence ratings.

Participants showed large differences in average response times (see Figure 3C), resulting in a wide range of prime/target stimuli durations. To examine whether the duration of the stimuli influenced the strength of the affective priming effect, a “priming effect sensitivity” (PES) score was computed for each participant. This was achieved by examining trials where two “Happy,” two “Angry,” or two “Fearful” faces were presented consecutively. For each instance where the participant rated the second face as even more positive (for “Happy” faces) or even more negative (for “Angry” or “Fearful” faces) than the first, a point was added to their PES score. Conversely, each time the participant rated the second face as less positive (for “Happy” faces) or less negative (for “Angry” or “Fearful” faces), a point was subtracted from their PES score. No points were added or subtracted if the participant gave the same rating to both faces. With only three exceptions, all participants showed a positive PES score (sign-test value of *p* = 0.000027). It is important to note that in the absence of any affective priming effect, most participants would likely yield a negative PES score due to boundary effects inherent in the 1–9 rating scale. Interestingly, we observed no relationship between the response time (which was directly correlated with the duration of the prime/target stimuli) and the strength of the affective priming effect (Figure 3C).

Table 1 display the mean participant ratings across blocks and primer-target conditions. While not always statistically significant, positive affective priming effects (indicated by green shading) were observed in all blocks when the prime and target had the same valence (either positive or negative). However, surprisingly, this effect was not present when the target was neutral. In fact, in some cases, affective priming effects were absent (blocks 2, 3, and 4) or even significantly negative (block 1) when the target was neutral.

To investigate whether priming effects were affected by the type of negative affect condition (“Fearful” in blocks 1, 3, and 5, or “Angry” in blocks 2 and 4), or by the orientation of the displayed faces (frontal view in blocks 1, 2, and 5, or half-profile view in blocks 3 and 4), or by the level of facial information available (whole face in blocks 1 to 4, or only eyes in block 5), a repeated measures ANOVA was conducted. The analysis did not reveal any significant differences in affective priming effects between the different blocks for targets with a negative valence. This same outcome was obtained when the analysis was repeated for targets with a neutral or positive valence.

## Discussion

The study demonstrates that the assessment of valence in consciously perceived images displaying facial emotional expressions, presented sequentially and easily recognized by participants (as shown in Figure 2 across the 5 blocks), is significantly affected by a robust affective priming effect (Figure 3A). However, this effect differs from those observed in studies utilizing subliminal and supraliminal primes to modulate the perception of neutral targets (Carroll and Young, 2005). In this study, no positive affective priming effect was observed for neutral targets, and for one block, a negative affective effect was even observed (see Table 1). Conversely, the valence of an emotional face was perceived as even more intense (either positive or negative) when preceded by a face with a similar expression, while the valence perception of a facial expression was perceived as more moderate (either less negative or less positive) when preceded by a face of opposite valence. One possible reason for the lack of attention to this effect in prevous studies is that priming effect research has typically focused on how a neutral target is perceived, rather than on targets with already strong valence.

Although most affective priming studies typically use subliminal primes, the experiments in this study were designed to imitate real-world situations. During social interactions with multiple individuals, people constantly shift their gaze from one face to another, and subtle affective priming effects are likely to take place continuously, simply by interacting with others. While the priming effects of words, objects, and actions on our behavior and perception have been well established (Weingarten et al., 2016), the results reported here suggest that similar effects also occur with emotional expressions. For instance, seeing two scared faces in succession in a real-life situation is likely to be associated with danger more strongly than when only one individual displays that facial expression. Similarly, seeing consecutive angry faces is more likely to be experienced as a threat to personal safety than when only one individual among many displays such an expression. Therefore, seeing consecutive emotionally valanced faces may be linked with activation of the autonomous nervous system. If enhanced valence perception and parasympathetic activation are linked, it would be interesting to identify the directionality of this causal relationship.

Cross-modal studies consistently show that subliminal facial expressions of different emotions can strongly affect how participants evaluate subsequent targets, such as Chinese ideographs (Murphy and Zajonc, 1993), words (Carroll and Young, 2005), pseudo speech with emotional intonation (Garrido-Vásquez et al., 2018), or even their behavior, such as pouring and consuming beverages (Winkielman et al., 2005) or making purchase decisions (Steffen et al., 2009). A leading theory to explain these effects is that participants are not exposed to the prime for long enough to process it consciously, and therefore misattribute the automatically experienced emotion to the target instead. This theory may also explain the effects observed in the current study: because the prime is presented for a sufficiently long time to process the emotional expression fully, the nature and intensity of the valence experience are correctly attributed to the prime. As a result, the response to the target is more intense when its emotional affect matches that of the prime. This is similar to the increased response time observed in semantic priming studies when the prime and target belong to the same semantic category (Klauer, 1997).

When participants rate a neutral face, they tend to select a value from the middle of the scale range, providing enough flexibility to measure shifts in perception in either direction. However, for faces with a positive or negative valence, they are more likely to be rated with a value from the extremes of the scale. As a result, targets cannot be rated more positively or negatively if maximum and minimum values are already used to rate the prime. These restrictions suggest that the reported effect’s intensity is likely conservative and potentially stronger than observed. Moreover, stimuli categorized as “neutral,” “negative,” or “positive” have subtle differences in arousal level among the emotional faces within the same affect category (Garrido and Prada, 2017), which can introduce noise. Randomly shuffling the trials presented to each participant helps average out these differences.

Each stimulus was presented for a minimum duration of 1 s and disappeared either in response to a button press, indicating the evaluation of the stimulus, or when the maximum display time of 4 s elapsed. Due to individual differences in response times, the average stimulus display times varied among participants, ranging from approximately 1.1–2.1 s. However, analysis of the data indicates that the strength of the observed priming effect did not vary within this range of stimulus duration (see Figure 3C).

Huang et al. (2018) found that subliminal face stimuli with an egocentric vantage point had a greater effect on the perception of angry faces compared to stimuli with an allocentric vantage point, suggesting that the priming effect is modulated by the relevance of the stimuli to the observer. Another study (Belopolsky et al., 2011) showed that participants were slower to shift their gaze away from an angry face compared to either a neutral or happy face. While the current study employed a block design (see “Stimuli and Experimental Conditions” and Figure 1) to probe different conditions (see Table 1), no evidence was found for an affective prime effect that is selective for the type of negative emotion or for the viewpoint or gaze direction. However, it is important to note that the absence of evidence does not necessarily imply evidence of absence. It is possible that the differences between the probed conditions are too subtle to detect with the limited sample size used in this study.

We acknowledge that our study has some limitations that may have impacted our results. Firstly, the KDEF dataset used in our study only consists of Caucasian faces, which limits the diversity of our sample. While other more diverse face databases are available, the KDEF dataset offers unique advantages as it includes models displaying various emotional expressions from different angles, including the straight and half frontal view used in this study. Secondly, all participants were young adults, which could be considered both a limitation and a strength. On one hand, age can be a variable that adds noise to the data, but on the other hand, having a fixed age group allows for a more controlled comparison. Lastly, the majority of our participants were female, which is not ideal as a more balanced gender distribution would have been preferable.

## Data availability statement

The raw data supporting the conclusions of this article will be made available by the author, without undue reservation.

## Ethics statement

The studies involving human participants were reviewed and approved by the IRB of Millsaps College. The participants provided their written informed consent to participate in this study.

## Author contributions

NB conceived of the conceptual idea, designed the experimental paradigm, contributed with the collection of the data, conducted all analyses, and wrote the manuscript.

## Funding

NB received grant support from the National Institutes of Health (NIH)/National Institutes of Neurological Disorders and Stroke (NINDS), R15NS121788.

## Conflict of interest

The author declares that the research was conducted in the absence of any commercial or financial relationships that could be construed as a potential conflict of interest.

## Publisher’s note

All claims expressed in this article are solely those of the authors and do not necessarily represent those of their affiliated organizations, or those of the publisher, the editors and the reviewers. Any product that may be evaluated in this article, or claim that may be made by its manufacturer, is not guaranteed or endorsed by the publisher.
